# Non-prescription treatments for childhood infections: an Austrian, monocentric, cross-sectional questionnaire study

**DOI:** 10.1186/s12887-022-03220-6

**Published:** 2022-03-24

**Authors:** Matthias Gerlitz, Peter Voitl, Julian J. M. Voitl, Susanne C. Diesner-Treiber

**Affiliations:** 1 First Vienna Pediatric Medical Center, Donau-City-Strasse 1, 1220 Vienna, Austria; 2 Sigmund Freud University Vienna, Donau-City Strasse 1, 1220 Vienna, Austria

**Keywords:** Adolescents, Children, Infections, Non-prescription treatments, Over-the-counter medication, Questionnaire

## Abstract

**Background:**

Infectious diseases like the common cold, otitis media, or gastroenteritis frequently occur in childhood. In addition to prescription drugs, parents often use supplementary over-the-counter (OTC) products recommended by pharmacists and other non-medical professionals to relieve their children’s symptoms. However, the efficacy of such alternative treatments lacks conclusive evidence.

The objective of this study was to investigate the use of OTC products and related active ingredients in children, and the motivations behind this choice.

**Methods:**

The present study included 215 children aged between 1 and 14 years with an acute respiratory tract infection, e.g., common cold, bronchitis, otitis media, tonsillitis, or gastroenteritis. During their visit to the pediatric practice, parents filled in a self-administered questionnaire about their child’s diagnosis, additional treatment options, and motivations to integrate supplementary medicinal products after their first visit for acute infection or follow-up examination. Children with chronic illnesses and patients visiting for a routine maternal and child health program check-up were excluded.

**Results:**

The study included 111 (51.6%) males and 104 (48.4%) females. Median age was 3.00 (IQR 2.0 – 5.0) years. The most common reason for a visit was a respiratory tract infection (78.6%). Out of 215 parents, 182 (84.7%) resorted to non-prescription remedies to alleviate their child’s symptoms. Teas (45.1%), and home remedies (43.3%) were the most popular. At total 133 (74.3%) followed recommendations from friends and family regarding additional medications usage. Parents with previous experience with complementary medicine tended to prefer this approach to treat their children (*p*.adjust = 0.08).

**Conclusion:**

The use of non-prescription medicine is increasing as well as the range of related information sources. Evidence-based recommendations in this field might improve pediatric care.

**Supplementary Information:**

The online version contains supplementary material available at 10.1186/s12887-022-03220-6.

## Introduction

The usage of additional medications as an adjunct treatment to formal medical care is a widespread phenomenon both among adults [1], and children [2–4], and the popularity of long-established home remedies, as well as non-prescription drugs available in pharmacies and on the internet seems to be undisputed. In Germany, the over-the-counter (OTC) market revenue increased by 1.4% in 2019 compared to the previous year, and so far, every second pharmaceutical sold in German pharmacies is an OTC drug [5]. The options range from synthetic chemical preparations and phytopharmaceuticals to homeopathy and anthroposophic medicine. In addition to the products currently on the market, self-medication can include different home remedies to attenuate the symptoms of common ailments.

The motivations behind this consumers' buying behavior can be manifold, but research in this field is still limited. Previous studies have indicated that, besides easily accessible information and the possibility to purchase anonymously online, gender, age, and socioeconomic status seem to be strong determinants [6].

These findings can be partially explained due to a higher awareness towards self-care among women [7] and a better knowledge of available pharmaceutical products among people with higher educational levels [8].

Although additional medications are being used broadly on children, data on frequency and efficacy are rare. A German survey including over 17,000 children and adolescents pointed out that 25.2% of all parents had used additional therapies in the week before the questionnaire, and, in this group, 17% had used OTC preparations. Usage was common among families with a higher income, and mothers with a higher education [9]. About 10% of US children were given cough or cold preparation in a given week [2].

Due to the wide range of OTC medications currently available, most countries regulate their active pharmaceutical ingredients. Antitussives [10–14], expectorant agents [15, 16], and antihistamines [17–19], are widely used and studies have proven the benefits of some of them in the symptomatic treatment childhood infections. Nevertheless, those substances are not suited for the treatment of the infection itself. In children, common ingredients like honey appeared to improve sleeping quality and act as a cough suppressant [20–23], while echinacea seemed to prevent respiratory tract infections [24]. Conversely, evidence on the use of garlic [25], Pelargonium sidoides extracts [26], and homeopathic remedies [27, 28] is more debatable. Generally, acknowledged randomized, placebo-controlled studies are rare, and while some substances can be associated with severe side effects and should be taken with caution [29, 30], their reported benefits remain under scrutiny.

This monocentric observational study aimed to give an overview of additional non-prescription medications used in acute childhood infections, investigate their distribution, and observe parents’ motivations regarding their usage.

## Methods

### Study design and population

This monocentric, cross-sectional study designed to include a self-administered questionnaire was approved by the Ethics Committee of the Medical University of Vienna (EK Nr: 2214/2018). All experimental protocols and procedures performed in this study involving human participants were carried out in accordance with the ethical standards of the Ethics Committee of the Medical University of Vienna and with the 1964 Helsinki declaration and its later amendments or comparable ethical standards. By filling in the questionnaire, all subjects and/or their legal guardians gave written informed consent to participate in this study. Eligible subjects were children aged 0–17 years, who visited the pediatric practice due to an acute infection, including the common cold, cough, bronchitis, tonsillitis, otitis media, or diarrhea. We included patients who came for primary care visits and follow-up appointments. Children with chronic illnesses or visiting for a routine maternal and child health program check-up were excluded from the study.

All participants were recruited at Vienna’s largest pediatric practice, “First Vienna Pediatric Medical Center “, and written informed consent was obtained from all the parents or guardians of the children. The authors made sure that the collected data could not identify specific patients or their attending physicians. A total of 250 participants were approached orally and asked to attend to the study. Of those, 18 (7.2%) immediately refused to participate (response rate: 92.8%). At least 90% of the questionnaire had to be completed for the participants to be included in this study. Therefore, 17 (7.3%) incompletely filled-in questionnaires were declined and not processed for further analysis.

### Questionnaire

The author designed the questionnaire according to international standards [31].All participants filled it in anonymously (Additional file 1). The first section included general demographic parameters such as age, gender, and education of the parents and children. The second section covered the use and motivations behind choosing to resort to non-prescription medications. Whenever possible, answers were organized on an ordinal scale (agree/ rather agree/ do not agree); if not, nominals (yes/ no) were used. The questionnaire contained the 15 items listed below:

General parameters:

- Age of the child
- Gender of the child
- Age of the parent
- Highest parental education

Special parameters:

- What is the doctor’s diagnosis?
- What is the treatment prescribed by the doctor?
- Do you think your doctor explained the prescribed treatment properly?
- Did you use an additional non-prescription treatment?
  o If yes, which one/s?
- Who recommended it to you?
- Do you think that the additional medication helped to reduce your child’s symptoms?
- Is your child sick a lot (more than 10x/year)?
- Do you have any experience with non-prescription treatments in your child’s siblings?
  o If yes, were those experiences positive?
- Are you using complementary remedies to relieve your symptoms?
- Did you follow your doctor's directions as prescribed?

### Statistics

The raw data was compiled using IBM SPSS Statistics (Version 25.0). Missing data were excluded from the calculation and indicated in the corresponding section. Statistical evaluation was conducted on the total number of participants. The presented data were observed over a fixed period of 6 weeks. All members of this study were recruited during this period and included into analysis. Therefore, no statistical power analysis was conducted prior to the start of the study. Parameters were described in categories (absolute, relative), and continua (mean, standard deviation, or median and IQR). Due to the limited sample size and the monocentric design of this study, the mentioned relative percentages only relate to the investigated cohort rather than the general population. The group that used additional therapies was compared to the one that did not. The χ2 test was performed to determine statistical significance for categorical variables. Fisher's exact test was applied for sample sizes < 5. Metric variables were tested using the Mann–Whitney-U Test, normal distribution was determined with the Shapiro–Wilk-Test. The two-sided *p*-value was set at ≤ 0.05. The phi-coefficient was used to observe the correlation between the various parameters. The Cramér's V coefficient was applied in the case of more than two variables. Odds ratios measured the strength of association. Correction for multiple testing was done using the Bonferroni-Holm method.

## Results

### Study population characteristics

A total of 215 children aged 1–14 years were included in this study; the median age was 3 years (IQR = 3; 2.0 – 5.0), 111 participants were male (51.6%), and 104 were female (48.8%). The mean age of parents was 34.09 years ranging from 19 and 53 years (SD = 5.96). Eighteen (8.4%) finished compulsory school, 47 (21.9%) served an apprenticeship, 69 (31.6%) graduated from high school, and 80 (37.2%) from college.

### Diagnoses and prescribed therapies

During the observation period, in total 169 (78.6%) children visited the doctor’s office due to an infection, i.e., a common cold, cough or bronchitis. Twenty-two (10.2%) were diagnosed with tonsillitis, 35 (16.3%) with otitis media, and 16 (7.4%) had diarrhea. Seven (3.3%) patients presented with symptoms that were not listed in the questionnaire i.e., three cases of urinary tract infections, two cases of scarlet, one case of constipation, and one of odontiasis. Some children suffered from more than one medical condition, with an average of 1.15 diseases diagnosed per child.

Regarding prescribed therapies, 158 (73.5%) participants received analgesics or antipyretics, 59 (27.4%) antibiotics, 61 (28.4%) inhalation therapy with sodium chloride, bronchodilating agents or glucocorticoids and 84 (39.1%) cough syrup. On average, the attending pediatrician prescribed 1.7 therapies per child.

### Additional therapies

One hundred eighty-two parents (84.7%) stated to use additional therapies to relieve their child’s symptoms, specifically home remedies, compresses, teas, homeopathic preparations, and other substances.

Ninety-three parents (43.3%) used *home remedies;* in this subgroup, 69 patients (74.2%) were diagnosed with a respiratory tract infection as a single diagnosis, 24 (25.8%) had comorbidities. The most traditional folk remedy was homemade onion syrup. Lastly, nine parents (10.8%) preferred honey. Other ingredients were ginger or lemon preparations (Table 1).

**Table 1:** Home remedies

Home remedies (HR)	Absolute frequency	Relative total frequency [%]	Relative frequency HR [%]	Relative cumulative frequency HR [%]
Onion	54	25.1%	65.1%	65.1%
Honey	9	4.2%	10.8%	75.9%
Lemon/Ginger	2	0.9%	2.4%	78.3%
Aroma-oil	2	0.9%	2.4%	80.7%
Soups	2	0.9%	2.4%	83.1%
Other	14	6.5%	16.9%	100.0%
Specification total	83	38.6%	100.0%	
HR total	93	43.3%		
No specification	122	56.7%		
TOTAL	215	100.0%		

Fifty-eight parents (27%) applied *compresses;* in this subgroup, 32 patients (55.2%) were diagnosed with a respiratory tract infection as their single diagnosis. Fifty-five (94.8%) described the kind of compress, 36 (65.5%) soaked it in vinegar, nine (16.4%) used it in combination with onion (Table 2).

**Table 2:** Compresses

Compresses	Absolute frequency	Relative total frequency [%]	Relative frequency compresses [%]	Relative cumulative frequency compresses [%]
Vinegar	36	16.7%	65.5%	65.5%
Onion	9	4.2%	16.4%	81.8%
Compress not specified	5	2.3%	9.1%	90.9%
Other	5	2.3%	9.1%	100.0%
Specification total	55	25.6%	100.0%	
Compresses total	58	27.0%		
No specification	157	73.0%		
TOTAL	215	100.0%		

Ninety-seven participants (45.1%) used various *teas*. In this subgroup, 66 (68%) received a single diagnosis of a respiratory tract infection. Ninety (92.8%) specified the type of preferred infusion, namely. 23 (25.6%) favored chamomile tea, 18 (20.0%) administered thyme tea, and 12 (13.3%) various cough and bronchitis soothing preparations (Table 3).

**Table 3:** Teas

Teas	Absolute frequency	Relative total frequency [%]	Relative frequency teas [%]	Relative cumulative frequency teas [%]
Chamomile	23	10.7%	25.6%	25.6%
Thyme	18	8.4%	20.0%	45.6%
Bronchial/Anti-cough	12	5.6%	13.3%	58.9%
Sage/Ribwort	10	4.7%	11.1%	70.0%
Herb	8	3.7%	8.9%	78.9%
Fennel	7	3.3%	7.8%	86.7%
Fruit	6	2.8%	6.7%	93.4%
Ginger	4	1.9%	4.4%	97.8%
Other	2	0.9%	2.2%	100.0%
Specification total	90	41.9%	100.0%	
Teas total	97	45.1%		
No specification	118	54.9%		
TOTAL	215	100.0%		

Fifty-one (23.7%) opted for *Homeopathic,* i.e. sea salt sprays, vitamin supplementations, red light therapy (RLT), or mineral salt preparations.

In children 2 to 6 years old, *home remedies* (46.3%) and *teas* (45.6%) were the most prominent used additional therapies. Older children, i.e. 7 to 12 years, tended to use *teas* (55.9%) as their main therapeutic agent (Table 4).

**Table 4:** Additional therapy usage according to age group

Age (years)	Number of participants	Home remedies		Compresses		Teas		Homeopathic	
		total	%	total	%	total	%	total	%
** < 2**	29	10	34.5%	6	20.7%	9	31.0%	3	10.3%
**2–6**	149	69	46.3%	39	26.2%	68	45.6%	39	26.2%
**7–12**	34	13	38.2%	12	35.3%	19	55.9%	8	23.5%
** > 12**	3	1	33.3%	1	33.3%	1	33.3%	1	33.3%
**TOTAL**	215	93	43.3%	58	27.0%	97	45.1%	51	23.7%

### Source of recommendation

One hundred seventy-nine (98.4%) participants made a statement about the source of recommendation, which included friends and family in 133 (74.3%) cases, 53 (29.6%) took advice from pharmacists, 47 (26.3%) from the internet, and 16 (8.9%) had other sources (i.e. other physicians, literature). On average, parents used suggestions from more than one source to decide about the additional treatment for their child.

### Additional therapy usage

Out of 182 parents (84.7%) who claimed to rely on supplementary therapies, 179 (98.4%) made a statement about the mitigation of their children’s symptoms. Only nine participants (5.0%) noticed the non-prescription remedies lead to no improvements in their child's physical well-being.

No significant statistical correlation emerged between the use of supplementary treatments and the following parameters: age of the child (*p* = 0.4); age of the parent (*p* = 0.8); sex of the child (*p* = 0.7); parental education (*p* = 0.1); adequate explanation of the prescribed treatment from the doctor (*p* = 0.2); frequency of illness episodes of the child (*p* = 0.5); parents' use of supplementary treatments (*p* = 0.2), and use of prescribed therapies (*p* = 0.3) (Tables 5 and 6).

**Table 5:** Self-medication—general parameters

	**TOTAL *n* = 215**	**Self-medication YES *n* = 182**	**Self-medication NO *n* = 33**	***P*-value**
**Self-medication YES**	182 (74,7%)	-	-	
**Age Child (years)**^**a**^	3 (2–5)	4 (3–6)	2 (1–4)	0.4
**Age Parent (years)**^**b**^	34 (30.5–38.0)	35 (31.0–38.5)	33 (30–37)	0.8
**Sex Child (female)**	104 (48.4%)	89 (85.6%)	15 (14.4%)	0.7
**Educational level**^**c**^	Compulsary school (18; 8.4%)	16 (88.9%)	2 (11.1%)	0.1
	Apprenticeship (47; 21.9%)	39 (83.0%)	8 (17.0%)	
	High school (68; 31.6%)	59 (86.8%)	9 (13.2%)	
	University (80; 37.2%)	68 (85.0%)	12 (15.0%)	

Data are described as n (%) for continuous as median (IQR)

Tests for significance are x^2^-test/ Fisher’s Exact Test (catergorial variables) or Mann – Whitney – U Test (continuous variables); *p* – value < 0.05 was considered significant

^c^*n* = 213, ^a^median and IQR, ^b^mean and SD

**Table 6:** Self-medication—special parameters

		**Total *n* = 215**	**Self-medication YES *n* = 182**	**Self-medication NO *n* = 33**	***P*-value (***p***.adjust)**^**a**^
**Explanation of the treatment**^**b**^	Do not agree	11 (5.2%)	10 (90.9%)	1 (9.1%)	0.2 (0.9)
	Rather agree	70 (33%)	63 (90%)	7 (10%)	
	Totally agree	131 (61.8%)	106 (80.9%)	25 (19.1%)	
**Frequent sickness of the child (more than 10x/year)**	Do not agree	159 (74%)	132 (83%)	27 (17%)	0.5 (0.9)
	Rather agree	36 (16.7%)	33 (91.7%)	3 (8.3%)	
	Totally agree	20 (9.3%)	17 (85.0%)	3 (15.0%)	
**Experience with treatment in siblings**	Yes	94 (43.7%)	86 (91.5%)	8 (8.5%)	**0.014 (0.084)**
	No	121 (56.3%)	96 (79.3%)	25 (20.7%)	
**Positive experience with siblings**^**c**^	Do not agree	6 (6.4%)	5 (83.3%)	1 (16.7%)	0.2 (0.9)
	Rather agree	40 (42.6%)	35 (87.5%)	5 (12.5%)	
	Totally agree	48 (51.1%)	46 (95.8%)	2 (4.2%)	
**Additional therapies used by parents**^**d**^	Do not agree	33 (15.4%)	26 (78.8%)	7 (21.2%)	0.2 (0.9)
	Rather agree	71 (33.2%)	57 (80.3%)	14 (19.3%)	
	Totally agree	110 (51.4%)	98 (89.1%)	12 (10.9%)	
**Use of prescribed therapies**^**e**^	Yes	205 (96.7%)	175 (85.4%)	30 (14.6%)	0.3 (0.9)
	No	7 (3.3%)	5 (71.4%)	2 (28.6%)	

Data for categorical variables described as n (%), tests for significance are x^2^-test/ Fisher’s Exact Test

^a^*p*-value was adjusted for multiple testing using Bonferroni-Holm method

^b^*n* = 212, ^c^*n* = 94, ^d^*n* = 214, ^e^*n* = 212

Our evidence emphasized a trendwise mild correlation between the usage of additional therapies and parents, who already had previous experience with various non-prescription medications on their other children (*p* = 0.014, *p*.adjust. = 0.084**,** Phi = 0.167) (Table 6). These parents were more likely to use home remedies or teas than parents without previous experiences (OR = 2.8; CI: 1.2–6.5).

It should also be noted that there was a mild but not significant correlation between parents who had previous and positive experience with additional therapies, although it did not show any significance (Cramér’s V = 0.163; *p* = 0.239) (Table 6).

## Discussion

This study aimed to assess parents' use of alternative medications on children with acute illnesses visited Vienna’s largest pediatric primary health care facility.

One hundred eighty-two (84.7%) interviewed parents stated they would use additional therapies to ease their child's sickness, although 95% of them were satisfied with their doctors’ diagnosis and prescribed medication. These figures contradict the assumption that parents would use complementary medicine only if unsatisfied with their doctors’ explanation about diagnosis or treatment.

The use of supplementary products in childhood infections turned out to be a widespread behavior in our study population (84.7%), but the principal sources for recommendations were friends and family, supposedly not all medical professionals. Pediatricians might find it challenging to advise due to the lack of randomized controlled trials on the substances, ingredients, and remedies mentioned in this study. Whereas there is data corroborating the effective cough suppressant action of honey in children older than two years [23], clinical trials investigating aromatherapy against respiratory tract pathogens are insufficient [32]. Similarly, there is no experimental evidence on the benefits of onion or ginger concoctions, as well as vinegar-soaked compresses.

Although many patients claimed teas do possess symptomatic relief properties against sore throat, no studies have produced clinical evidence on the any anti-inflammatory effects of different herbs [33, 34].

Until randomized control studies on homeopathic remedies prove the effectiveness of alternative medications, physicians should not recommend this practice [27, 28, 35].

This study did not investigate the side effects of the various preparations. Nevertheless, clinicians should raise awareness on the potential risks such as overdosage, possible adverse reactions, and interactions [29, 30, 36].

Although data on most remedies are rare or invalid, 95% of all included parents affirmed that additional therapies reduced their children’s symptoms. Confounding factors such as the self-limiting diseases, regression to the mean, and the action of prescription medications could have contributed to the positive attitude towards alternative medicine. Previous studies have also mentioned the placebo effect of OTC medication on children [16]. Nevertheless, as the patients' mean age in this study was three years, most children were not able to communicate an improvement in their symptoms, and the parents described the success of supplementary medications according to their observations.

Surprisingly, only 25% of all participants used medications recommended by pharmacists or from the internet, despite the direct advertising, especially during winter. According to our study recommendations friends and family remain the most common source to obtain information on what medication to use. Accordingly, the largest subgroup of additional therapies were home remedies that are not marketed, but handed down through word-of-mouth, meaning that traditional knowledge plays a crucial role.

Contrary to a large German study [9] in which about 25% of all surveyed parents used additional therapies on their children, the prevalence in this study was higher (84.7%). This discrepancy may depend on our decision to investigate the behavior of parents of sick children who, consequently, integrate supplementary products more often.

As opposed to the study mentioned above, we could not confirm the correlation between higher education and increased use of additional therapies, which might be caused by the small patient number in our study.

Considering the self-limiting nature of some diagnosed diseases and the principles of conservative prescribing, the number of prescribed antibiotics in our cohort (27.4%) appears relatively high. Emblematically, 17.8% of all respiratory tract infections required antibiotics. Due to the design of the questionnaire, we could not determine if antimicrobial agents were only prescribed in case of a bacterial infection such as pneumonia or if physicians ordered it to tackle viral bronchitis as well and actively contravened the guidelines. The elevated number of antibiotics prescribed against diarrhea (25.0%) might depend on the co-occurrence of a respiratory tract infection in 89% of those patients.

The main limitation of this study was the small sample size, explaining some of the non-significant results. Larger subgroups might generate different and statistically significant results. Because of the monocentric design of this study, the compiled data cannot lead to any conclusions on the Austrian general population. As the questionnaire was administered at the practice, parents' answers about their satisfaction with their attending physician might be biased.

The questionnaire itself may have been too complex for some of the participants; therefore, 10% of all questionnaires required clarifications, which might have altered the overall results.

Additionally, there might be differences between the "first visit" group and the "follow-up" one. For instance, some parents might have introduced a complementary product only after the first appointment. Therefore, separate investigations on these two groups might yield more accurate results.

Ultimately, parents who disagreed with the doctor’s prescription therapy and preferred additional therapies may have missed the follow-up check and could not be included in this study.

To the best of our knowledge, this monocentric, cross-sectional study is the first to present such data from an Austrian pediatric cohort, and comparable research in Austria is rare. Our work gives an overview about usage of additional therapies, sources of recommendation and reinforcing factors on using them. Nevertheless, further studies in larger cohorts and different primary health care facilities should be conducted to put those findings into perspective and to broaden our knowledge on parents' use of alternative medications on children.

## Conclusion

The use of non-prescription treatments for children's illnesses is widespread. Home remedies such as infusions and ingredients like onion or honey are only a few among the numerous alternatives. Typically, recommendation sources are friends and family, and parents with previous experience with non-prescription products on their other children rely on this approach more often than parents without previous experience.

Pediatricians and pharmacists should, whenever possible, provide evidence-based recommendations regarding the use of OTC products and counseling on complementary medicine and home remedies.

## Supplementary Information

**Additional file 1. **

## Data Availability

The data that support the findings of this study are available from the First Vienna Pediatric Medical Center but restrictions apply to the availability of these data, which were used under license for the current study, and so are not publicly available. Data are however available from the authors upon reasonable request and with permission of the First Vienna Pediatric Medical Center.

## Abbreviations

CI: Confidence interval
HR: Home remedies
IQR: Interquartile range
OR: Odds ratio
OTC: Over-the-counter
SD: Standard deviation
