# Supplementary material for: Non-prescription treatments for childhood infections: an Austrian, monocentric, cross-sectional questionnaire study
Source: BMC Pediatr. 2022 Mar 24;22:154. doi: 10.1186/s12887-022-03220-6 (PMC8943939; doi:10.1186/s12887-022-03220-6)
Supplement: Supplementary file 1 — Additional file 1. [file 12887_2022_3220_MOESM1_ESM.pdf]

Questionnaire

Non-prescription Treatments for Childhood Infections

General Parameters

Study number of the participating child ______________________________
(filled out by the investigator)

Age of the child ________________________________

Sex of the child

○ male ○ female

Age of the parent _________________________

Highest completed education of the parent

○ Compulsory school ○ Apprenticeship

○ High school ○ University

Special Parameters

What is the doctor`s diagnosis?

○ Infection of the respiratory tract (e.g., common cold, cough, bronchitis)

○ Inflammation of the tonsils (Angina tonsillaris)

○ Inflammation of the middle ear (Otitis media)

○ Dysentery (diarrhea)

○ Other diagnosis: ____

What therapy got prescribed by the doctor?

○ Painkillers and Antipyretics (e.g., Nureflex®, Benuron®, Mexalen®, Parkemed®)

○ Antibiotics (e.g., pills, sirups, ointments etc.)

○ Inhalation therapy (e.g., Sultanol®, sodium chloride, Flixotide®)

○ Cough sirup

Do you think your doctor explained the prescribed treatment properly?

○ I do not agree ○ I rather agree ○ I agree

Questionnaire

Non-prescription Treatments for Childhood Infections

Did you use any additional therapy, which was not prescribed by your physician, to treat the sickness of your child?

○ Yes ○ No

If yes, which ones?

○ Home remedies: Which ones? ____

○ Compresses: Which ones? ____

○ Teas: Which ones? ____

○ Homeopathics

○ Others: ____

Who recommended this therapy to you? (multiple answers possible)

○ Pharmacy ○ Friends/family ○ Internet

○ Others: ________________________________

Do you think that the additional medication helped to reduce the symptoms of your child?

○ I do not agree ○ I rather agree ○ I agree

Does your child get sick often (more than 10x/year)?

○ I do not agree ○ I rather agree ○ I agree

Do you have any experience in treatment with additional therapies in other siblings of your child?

○ Yes ○ No

If yes, were those experiences positive?

○ I do not agree ○ I rather agree ○ I agree

If sick, are you using additional therapies yourself to relieve your symptoms?

○ I do not agree ○ I rather agree ○ I agree

Questionnaire

Non-prescription Treatments for Childhood Infections

Did you use the doctor`s therapy as prescribed?

○ Yes ○ No

If no, why not?

____

____
